# Supplementary material for: Reliable bi-functional nickel-phosphate /TiO2 integration enables stable n-GaAs photoanode for water oxidation under alkaline condition
Source: Nat Commun. 2023 Sep 5;14:5429. doi: 10.1038/s41467-023-41120-0 (PMC10480475; doi:10.1038/s41467-023-41120-0)
Supplement: Supplementary file 1 — Supplementary Information [file 41467_2023_41120_MOESM1_ESM.pdf]

Supplemental Information

Reliable bi-functional Ni-Pi/TiO_2 integration enables stable n-GaAs photoanode for water oxidation under alkaline condition

Maheswari Arunachalam[1], Rohini Subhash Kanase[2], Kai Zhu[3*], Soon Hyung Kang[1*]

[1]Department of Chemistry Education and Optoelectronic Convergence Research Center, Chonnam National University, Gwangju 61186, Republic of Korea

[2]Department of Interdisciplinary Program for Photonic Engineering, Chonnam National University, Gwangju 61186, Republic of Korea

[3]Chemistry and Nanoscience Center, National Renewable Energy Laboratory, Golden, CO 80401, USA

*E-mails: Kai.Zhu@nrel.gov (Kai Zhu); skang@jnu.ac.kr (Soon Hyung Kang)

Supplementary Results and Discussion

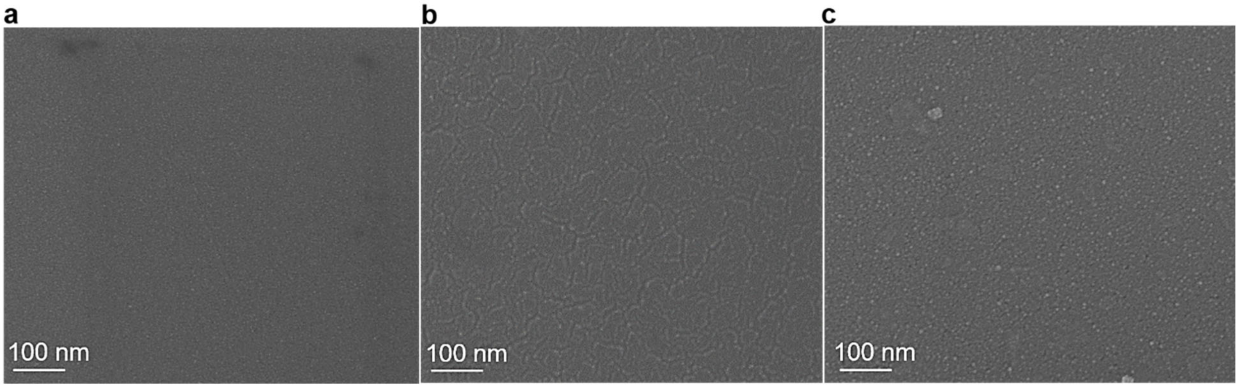

Supplementary Figure 1. Surface FE-SEM views of etched GaAs films (a) 10 min, (b) 20 min, and (c) 30 min.

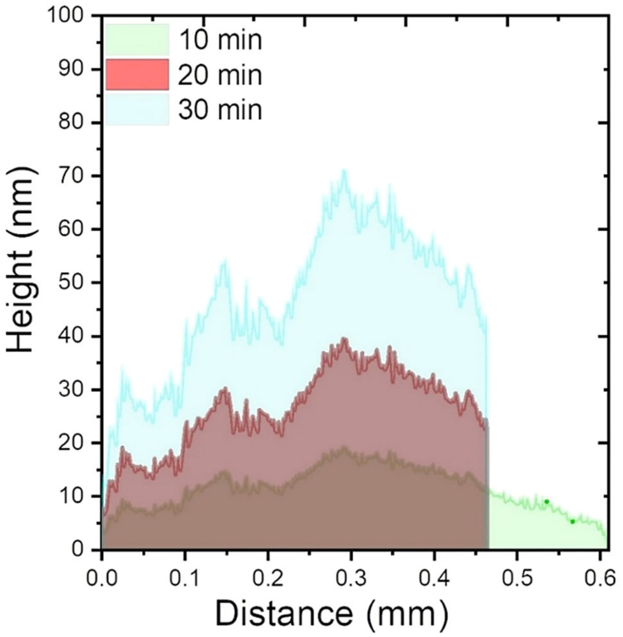

Supplementary Figure 2. Alfa-step depth profiles of the etched GaAs photoanode film with the etched time.

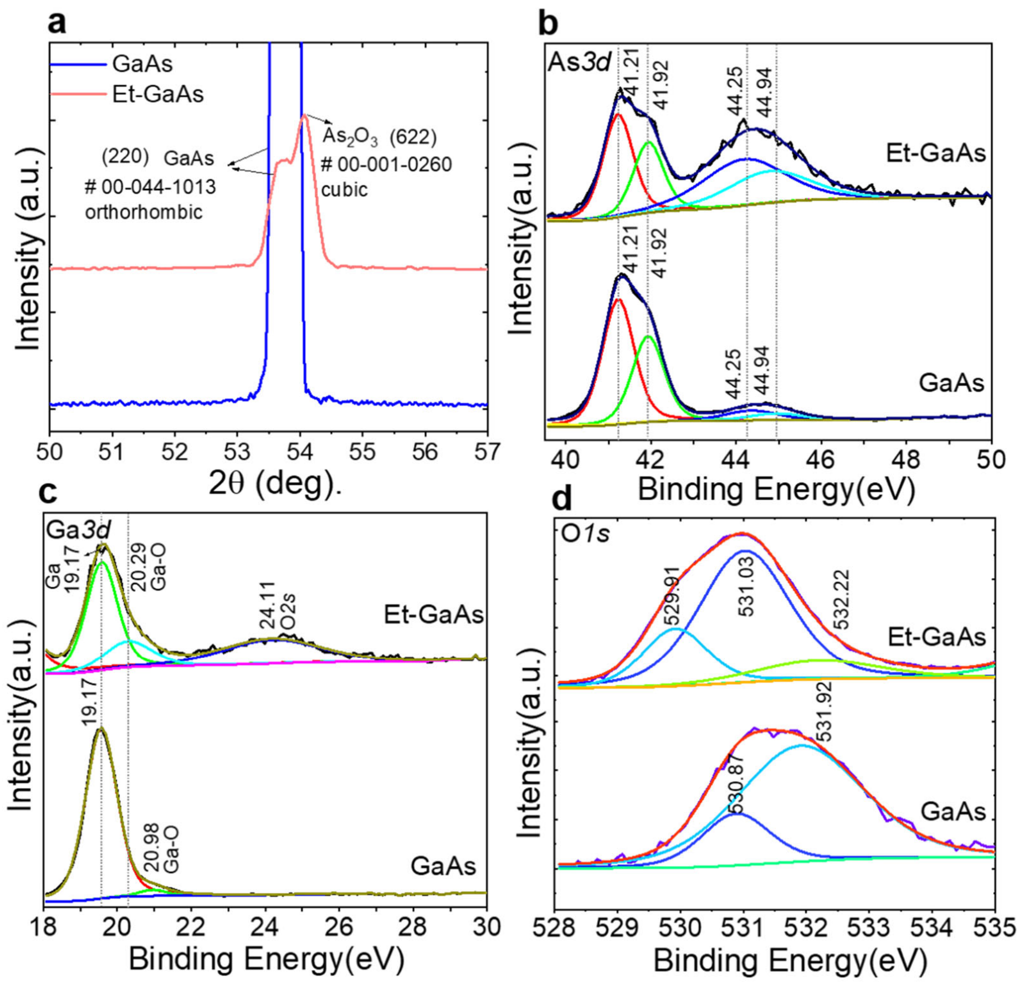

Supplementary Figure 3. (a) XRD patterns of GaAs and Et-GaAs films and (b–d) core-level XPS spectra of As*3d*, Ga*3d*, and O*1s* elements in GaAs and Et-GaAs films.

Supplementary Fig. 3(a) shows the XRD analysis results, confirming the native oxides generated during the etching process. Bare GaAs exhibits the orthorhombic crystalline structure, and the main diffraction peak is located at 53.75°, assigned to the (220) plane, according to International Centre for Diffraction Data (ICDD) file number (00-044-1013). Et-GaAs film exhibits the diffraction peaks of (220) and (622) planes, corresponding to the orthorhombic structured GaAs and cubic structured As_2O_3 phases, respectively. In order to understand the chemical state of the constituent elements of As*3d*, Ga*3d*, and O*1s* in the bare and Et-GaAs, X-ray photoelectron spectroscopy was analyzed, displayed in Supplementary Fig. 3(b–d). Supplementary Fig. 3(b) shows the core-level As*3d* spectra of as-received GaAs and Et-GaAs photoanode films. The As*3d* peaks of the as-received GaAs film at binding energies of 41.23 eV, 41.93 eV, and 44.71

eV can result from arsenic bonded to gallium (Ga-As), metallic arsenic (As^0), and small amount of surface native oxides (As_2O_3 and As_2O_5), respectively.[1] For the Et-GaAs film, the peaks at 41.5 eV and 45 eV can be deconvoluted into four key components, including As-Ga, As^0, and O-As-O (As_2O_3(As^{+3}) and As_2O_5(As^{+5})) bonding.[2] It is further calculated quantitatively by the integral area of the deconvoluted peaks. The area of As_2O_3(+3) is higher than As_2O_5(+5) in both the bare and ET-GaAs films. The calculated portion of each oxide is [5%(+3), 35%(+3)] and [1.8%(+5), 27%(+5)] for As_2O_3 and As_2O_5, respectively, in both photoanode films. Thus, the greater increase of the arsenic oxides at the Et-GaAs film is clearly evident during electrochemical etching. In particular, the position of each component being the same as in the pristine GaAs film demonstrates that the electrochemical etching process has no influence on the intrinsic nature of the GaAs, whereas the surface morphology of the films was largely modified. Further, the Ga*3d* spectra (Supplementary Fig. 3(c)) of the GaAs and Et-GaAs films are fitted with multiple peaks, and the peak of Ga$3d_{5/2}$ at binding energy 19.17 eV comes from the Ga metal in GaAs. Also, the shoulder at the binding energy of 20.98 eV corresponds to native Ga-O in the GaAs surface.[3]

Meanwhile, in the case of Et-GaAs film, the same binding energy at 19.17 eV was observed from Ga$3d_{5/2}$, but the significant variation from the shoulder peak was observed in the binding energy of 20.29 eV, due to the native gallium oxide (Ga-O). Next, in the case of the O*1s* spectra (Supplementary Fig. 3(d)), the pure GaAs and Et-GaAs are well fitted with the resolved peaks. The peaks at binding energies of 530.87 eV and 531.92 eV are attributed to native oxides, such as Ga-O and As-O, and non-lattice oxygen in bare GaAs film. The peaks at binding energies of 529.91 eV, 531.03 eV, and 532.22 eV are attributed to lattice oxygen in As_2O_3, Ga-O, or As-O and non-lattice oxygen, ascribed to metal bounded hydroxides in Et-GaAs film. This confirms the formation of a native oxide layer in the etched GaAs film during the electrochemical etching process.

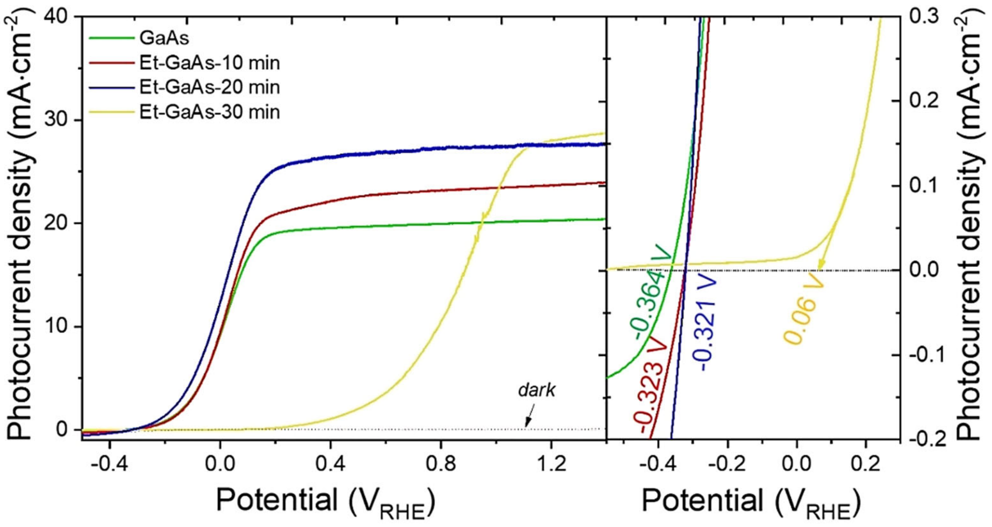

Supplementary Figure 4. LSV curves of GaAs, Et-GaAs-10 min, 20 min, and 30 min photoanode film with the magnified onset potential in the right part.

The GaAs photoanodes are etched for 10, 20, or 30 minutes at a constant current of 0.001A and are labeled as Et-GaAs-10 min, Et-GaAs-20min, and Et-GaAs-30min, respectively, shown in Supplementary Fig. 4. The obtained the onset potential and photocurrent density (J_{ph}) at 0 and 1.23 V_{RHE} of each photoelectrode as following Table 1. Based on the high J_{ph} and reproducible results in PEC condition, we take the Et-GaAs-20min photoanode for the further surface engineering. These results demonstrate that the surface etching of the photoanode film is a viable approach to modify the PEC behavior of GaAs-based photoelectrodes.

Supplementary Table 1. Summary of onset potential, J_{ph} at 0 and 1.23 V_{RHE} of GaAs, Et-GaAs-10 min, Et-GaAs-20min, and Et-GaAs-30min films, respectively

	onset potential (V_{RHE})	J_{ph} at 0 V_{RHE}	J_{ph} at 1.23 V_{RHE}
GaAs	-0.364	10.21	20.3
Et-GaAs-10min	-0.323	11.1	23.61
Et-GaAs-20min	-0.321	13.5	27.38
Et-GaAs-30min	-0.060	0.11	28.29

The etched samples show the positive shift of onset potential, compared to that of bare GaAs film, closely related to the initial surface kinetic barrier, due to the formation of native oxide during the etching process. In addition, the LSV curve has the same shape for the Et-GaAs-10min and 20

min, in which the difference of J_{ph} can be explained by the depth of surface etching sufficiently affecting the surface features such as porosity, roughness and surface area etc. Conversely, the complete opposite trend is remarkably noticed on the Et-GaAs-30min film, coming from the high density of surface native oxides and totally impeding the charge transfer kinetics at the interface.

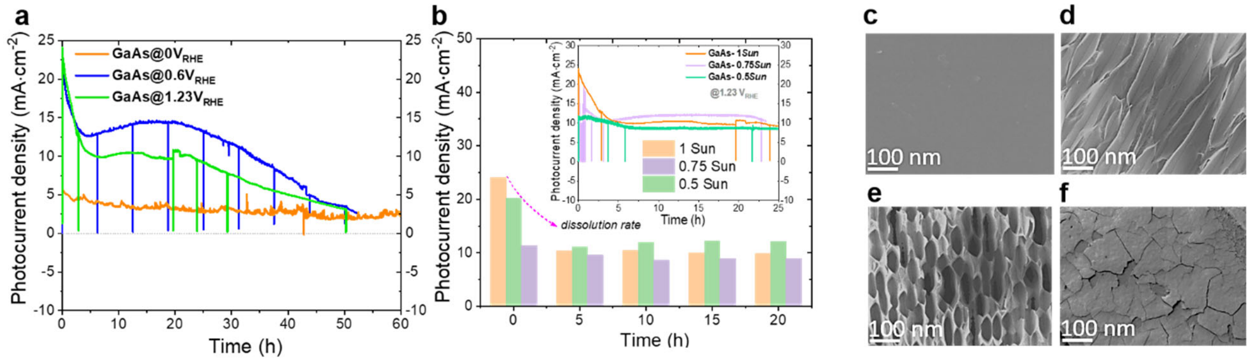

Supplementary Figure 5. (a) Chronoamperomentry test of bare GaAs film under different applied potentials of 0 VRHE, 0.6 VRHE, and 1.23 VRHE, respectively. (b) Dissolution rate versus different light intensities, with the inset showing the chronoamperomentry test of different light intensities (1 sun, 0.75 sun, and 0.5 sun) at a fixed applied potential of 1.23 VRHE. (c–f) FE-SEM images of photoanode films taken after long-term stability testing at different applied potentials of bare, 0 VRHE, 0.6 VRHE, and 1.23 VRHE, respectively, as shown in (a).

Supplementary Fig. 5(a) shows the original stability results of the GaAs electrode measured in a quartz cell, under 1M NaOH at different applied potentials of 0, 0.6, and 1.23 VRHE. The photocurrent decay is observed in the first 5 h of the stability testing at 0.6 and 1.23 VRHE, and the pronounced decay is 61% and 51%, respectively. Meanwhile, the observed decay at 0 VRHE is about 72%, proving that the bare GaAs corrosion rate depends highly on the applied potential and that the GaAs is a sensitive material under PEC working conditions. Similarly, the dissolution rate as a function of the prolonged time (25 h and 50 h) was summarized in Supplementary Fig. 5(b), revealing that the dissolution of GaAs in low potential is slower, middle potential is moderate, and high potential is extreme. However, after 50 h of testing, all amperometric (*i-t)* curves reached the same value, and GaAs completely detached from top to bottom. To understand the dissolution mechanism in depth, the stability test was carried out at different light intensities at a constant potential of 1.23VRHE. The inset of Supplementary Fig. 5(b) shows the GaAs dissolution rate under 1-, 0.75-, and 0.5-sun illumination, respectively. A lower light intensity (0.5-sun) led to better stability up to ~25 h, whereas a faster dissolution rate was observed under 1-sun and 0.75-sun intensity. The lower intensity leads to limited PEC performance due to the deficient energy carrier separation and increased kinetic overpotential for the water oxidation. Also, the surface morphology and structure of the bare GaAs film after the long-term stability testing at different applied potentials of 0, 0.6, and 1.23 VRHE were also probed and are displayed in Supplementary Fig. 5(c–f). A strong alkaline electrolyte with a high potential to induce some cracked or rougher

corrosion on its surface of the photoanode films. Typically, the formation of an insulating interfacial native oxide layer leads to pit formation that can spread through the entire photoelectrode in direct contact with electrolyte. In addition, a thick native oxide layer was identified under severe applied potential (Supplementary Fig. 5(f)). Therefore, it can be clearly understood that the photostability of a GaAs photoelectrode depends critically on both the light intensity and the applied potential under the strong alkaline electrolyte. These intriguing findings prompted us to explore the intrinsic origin of degradation for the durable PEC performance of the bare GaAs. Nanostructured surfaces offer high light absorption, high surface area, and other favorable operating properties. This means that an appropriate protecting layer can be recommended to suppress the GaAs dissolution process during the PEC reaction. In this work, we introduce crystalline TiO_2 as a protecting layer on the GaAs surface. Furthermore, the modulation of the surface nanostructure by the electrochemical etching process of bare GaAs film can improve the interfacial properties, such as the formation of thin native oxides partially taking action on the protecting layer, the high surface area/light absorption, and the strong contact between GaAs and TiO_2 materials (closely correlated to the charge transfer phenomenon). Under a well-controlled etching process, the synergistically effective GaAs surface can boost the interfacial properties under PEC working conditions.

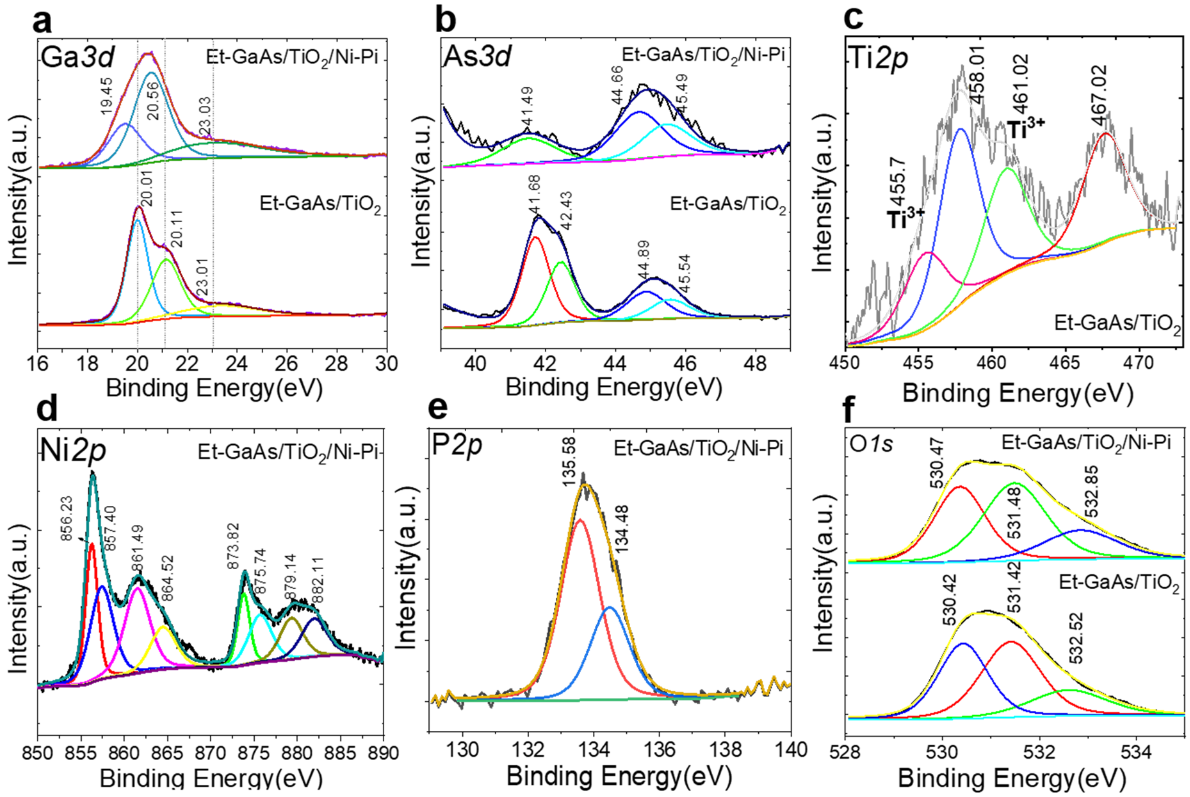

Supplementary Figure 6. (a–f) Core-level XPS spectra of Ga*3d*, As*3d*, Ti*2p*, Ni*2p*, P*2p*, and O*1s* of Et-GaAs/TiO_2 and Et-GaAs/TiO_2/Ni-Pi films.

Supplementary Fig. 6(a–f) shows the core-level XPS spectra of Ga*3d*, As*3d*, Ti*2p*, Ni*2p*, P*2p*, and O*1s* of Et-GaAs/TiO_2 and Et-GaAs/TiO_2/Ni-Pi films. In the case of Ga*3d* spectra (Supplementary Fig. 6(a)), the multiple peaks of binding energy at 20.1, 20.11, and 23.01 eV and 19.45, 20.56, and 23.03 eV from Et-GaAs/TiO_2 and Et-GaAs/TiO_2/Ni-Pi films can be deconvoluted, representing the metallic Ga, Ga-O, and O*2s* bonding environments, respectively. A peak shift is observed due to the interaction of multiple valence elements, and they are very tightly coupled.

Supplementary Fig. 6(b) shows the As*3d* spectra of Et-GaAs/TiO_2 and Et-GaAs/TiO_2/Ni-Pi photoanode films. The Et-GaAs/TiO_2 surface shows peaks due to arsenic bonded to gallium (Ga-As), metallic arsenic (As^0), and small amounts of surface native oxides (As_2O_3 and As_2O_5), at binding energies of 41.68, 42.43, 44.89, and 45.54 eV, respectively. For Et-GaAs/TiO_2/Ni-Pi, the peaks at 41.49, 44.66, and 45 eV can be deconvoluted into three key components, including As-Ga, As^0, and O-As-O ($As_2O_3(As^{+3})$, $As_2O_5(As^{+5})$) bonding. The negative shift of the As*3d* peaks

at the Et-GaAs/TiO_2/Ni-Pi films may be from the reduction of native oxides resulting from the negative applied potential during the Ni-Pi electrodeposition. In Supplementary Fig. 6(c), the Et-GaAs/TiO_2 and Et-GaAs/TiO_2/Ni-Pi films exhibited the +4 oxidation state related to Ti$2p_{3/2}$ and Ti$2p_{1/2}$, positioned at binding energies of 459.31 eV, 458.56 eV and 465.06 eV, 464.70 eV, respectively, and the +3 oxidation state related to Ti$2p_{1/2}$ is positioned at binding energies of 458.0 eV and 457.9 eV, respectively. The shifts happen via the addition of Ni-Pi around the host Ti ions. This augmentation in the area of the Ti^{3+} peak signifies that either a large amount of Ti_2O_3(+3) is formed, or that some mixed oxide structure of Ti-O-Ni is formed after co-catalyst deposition.[4,5] These defect states in the crystalline TiO_2 are mainly responsible for the hole transportation toward the surface to drive the significant PEC water oxidation. To understand the quantitative generation of oxygen vacancies (O_v), electron paramagnetic resonance (EPR) or electron spin resonance (ESR) spectroscopy is recorded to survey the presence of oxygen vacancies or Ti^{3+}, with paramagnetic species containing unpaired electrons. Here, Et-GaAs/TiO_2/Ni-Pi photoanode films were prepared under different annealing temperature conditions, and their EPR characters were compared at room temperature, as shown in Supplementary Fig. 7. Then, all films, including GaAs, Et-GaAs/TiO_2 (250°C)/Ni-Pi, Et-GaAs/TiO_2 (300°C)/Ni-Pi, and Et-GaAs/TiO_2(350°C)/Ni-Pi, showed strong resonance signals. Considering that the intensity variation of the EPR signal represents the presence of different magnitudes of oxygen vacancies, the EPR signal of Et-GaAs/TiO_2 (300°C)/Ni-Pi film exhibits a more intense and broader peak than the others. The most intense peak, with *g* factor at 2.002 and 1.983, implies that O_v and electrons trapped on oxygen vacancies were closely associated with Ti^{3+} on oxygen vacancies. The results demonstrate that O_v at the Et-GaAs/TiO_2-300°C film possesses the optimum concentration, contributing to the superior PEC performance (Figure 4(a)). Furthermore, the core-level Ni$2p$ spectrum of the Et-GaAs/TiO_2/Ni-Pi sample shows the BE peaks at 856.23, 857.40, 873.82, and 875.74 eV, consistent with those of Ni$2p_{3/2}$ and Ni$2p_{1/2}$ in $Ni_3(PO_4)_2$, assigned to the two spin-orbit doublets characteristic of Ni^{2+} and Ni^{3+} and two shakeup satellite peaks (labeled as Sat. 1 and Sat. 2). The peaks can interact with phosphate and hydroxide ions. In addition, the higher binding energy of 857.40 and 875.74 eV in the Ni$2p$ spectra can be assigned to the core-level peaks of Ni^{2+} cations, indicating the presence of $Ni(OH)_2$ in the hydrated $Ni_3(PO_4)_2$ structure.[6,7] The core-level P$2p$ spectra reveals the binding energy peaks at 134.48 and 135.58 eV, which were well fitted to $2p_{1/2}$ and $2p_{3/2}$ doublets, as displayed in Supplementary Fig. 6(e), ascribed to interaction of the P–O bonding in

$Ni_3(PO_4)_2$. In particular, the main deconvoluted peak (P^{5+}) at 134.48 eV constitutes 40% of the total area of the P*2p* spectrum in the Et-GaAs/TiO_2/Ni-Pi sample, indicating that PO_4^{3-} is the major component on the $Ni_3(PO_4)_2$ surface.[8,9,10] Additionally, the core-level O*1s* spectrum (Supplementary Fig. 6(f)) further confirms the formation of nickel phosphate. The core-level O*1s* spectrum exhibits one broad peak, which can be deconvoluted into three peaks at 530.77, 530.7, and 532.83 eV, which are ascribed to the lattice oxygen in the form of (GaAs)–O, P–O, and $Ni(OH)_2$ or adsorbed water molecules (H_2O), respectively. The strong and intense peak at 531.2 eV is closely associated with the Ni–OH and P–O bonds. The small intensity peak, positioned at 532.8 eV, occurs primarily from hydrates and moisture.[11]

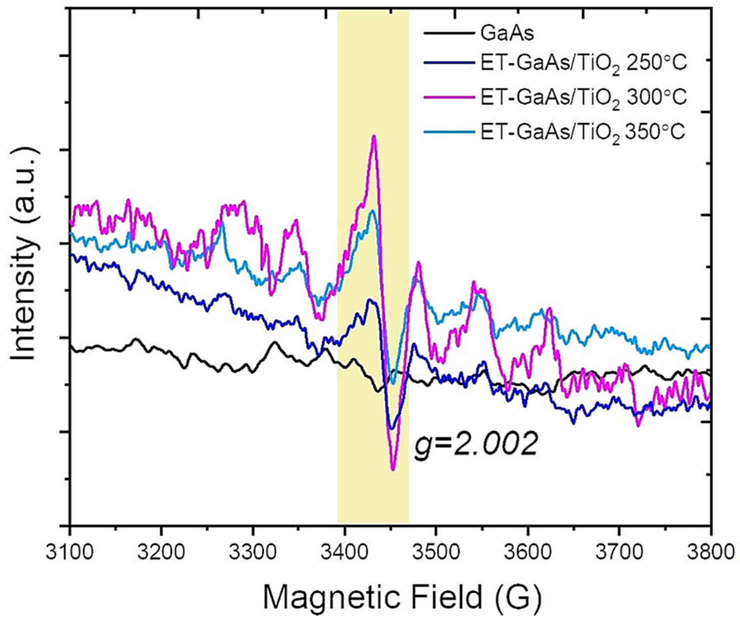

Supplementary Figure 7. Electron paramagnetic resonance (EPR) of GaAs, Et-GaAs/TiO_2 (250°C)/Ni-Pi, Et-GaAs/TiO_2 (300°C)/Ni-Pi, and Et-GaAs/TiO_2 (350°C)/Ni-Pi films.

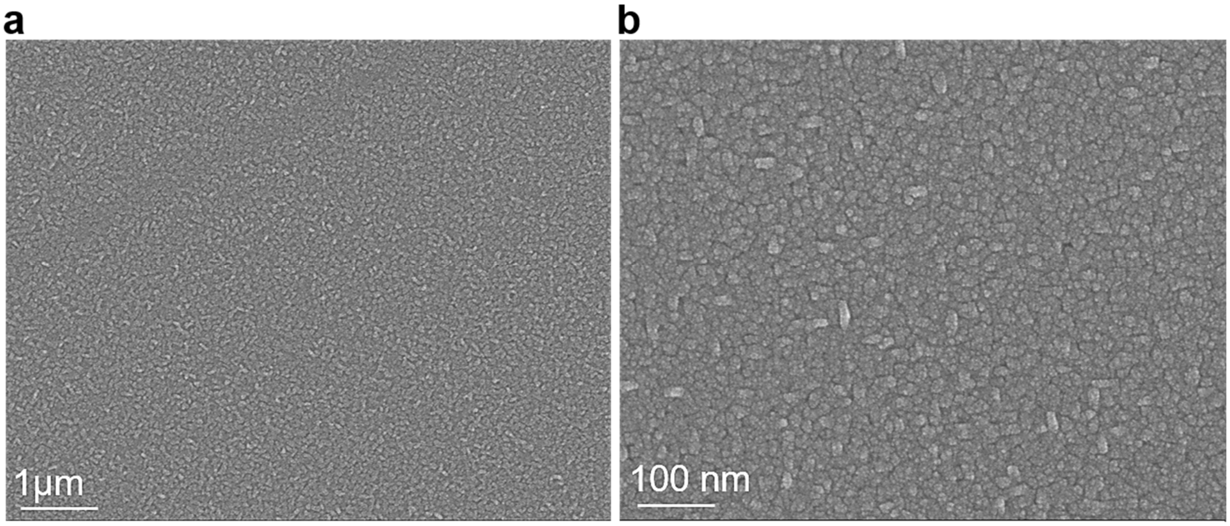

Supplementary Figure 8. (a-b) Surface FE-SEM views of Et-GaAs/TiO_2 film.

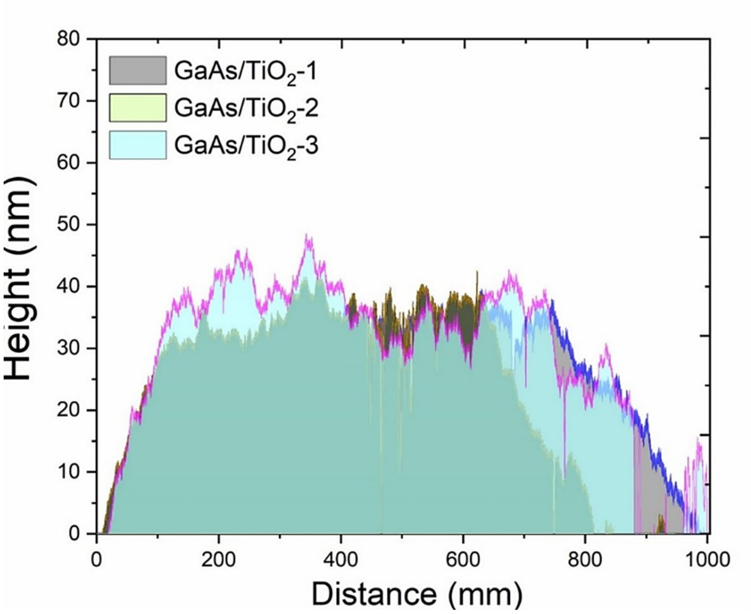

Supplementary Figure 9. Alpha-step profiles of planar GaAs/TiO_2 film.

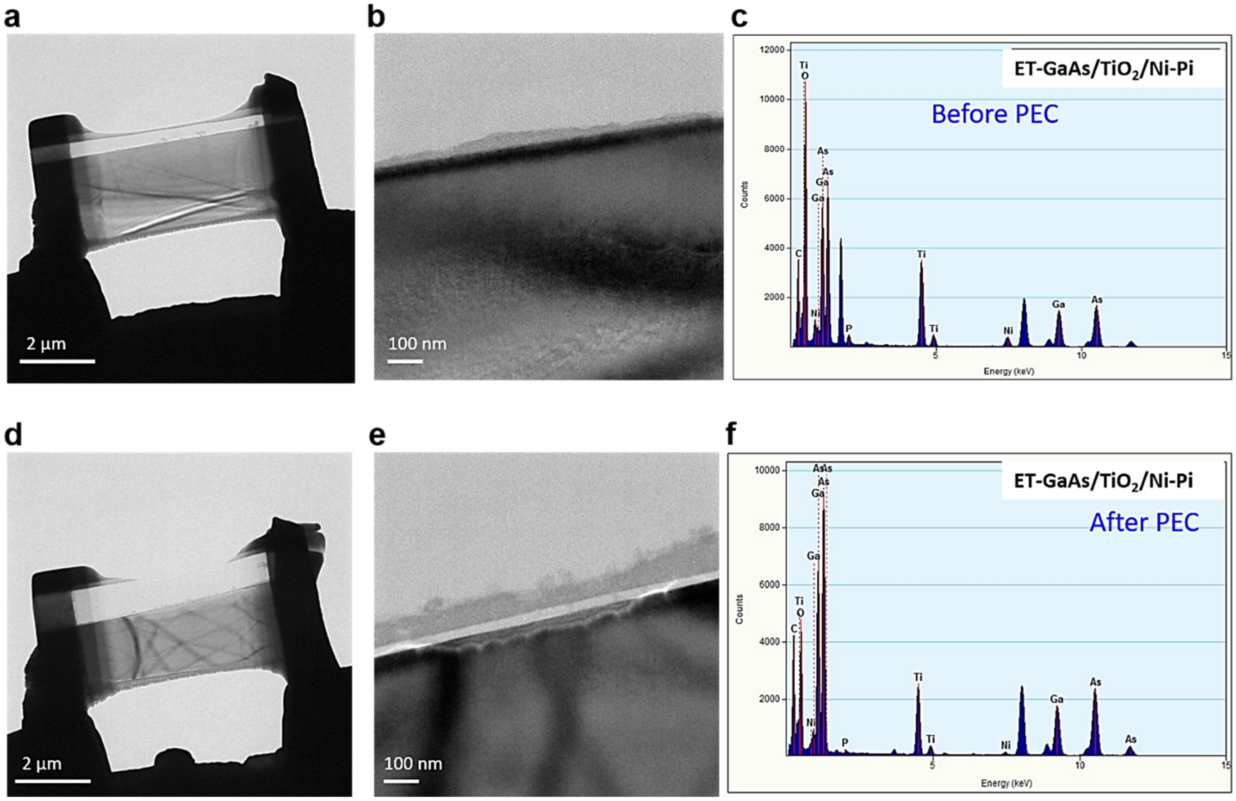

Supplementary Figure 10. (a–b) Low magnification of FIB-TEM images. (c) EDX spectrum of Et-GaAs/TiO_2/Ni-Pi before stability. (d–e) Low magnification of FIB-TEM images. (f) EDX spectrum of Et-GaAs/TiO_2/Ni-Pi after stability.

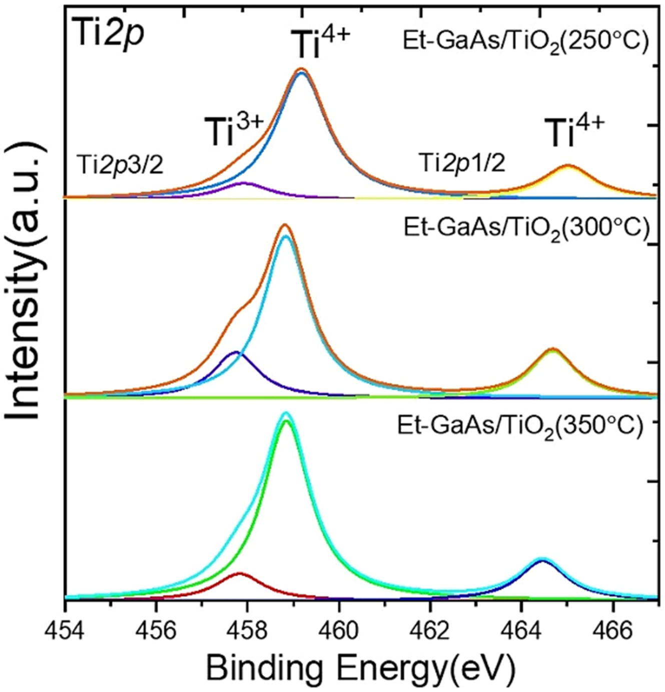

Supplementary Figure 11. (a) Core-level XPS spectra of Ti*2p* of Et-GaAs/TiO_2-250°C, Et-GaAs/TiO_2-300°C and Et-GaAs/TiO_2-350°C films.

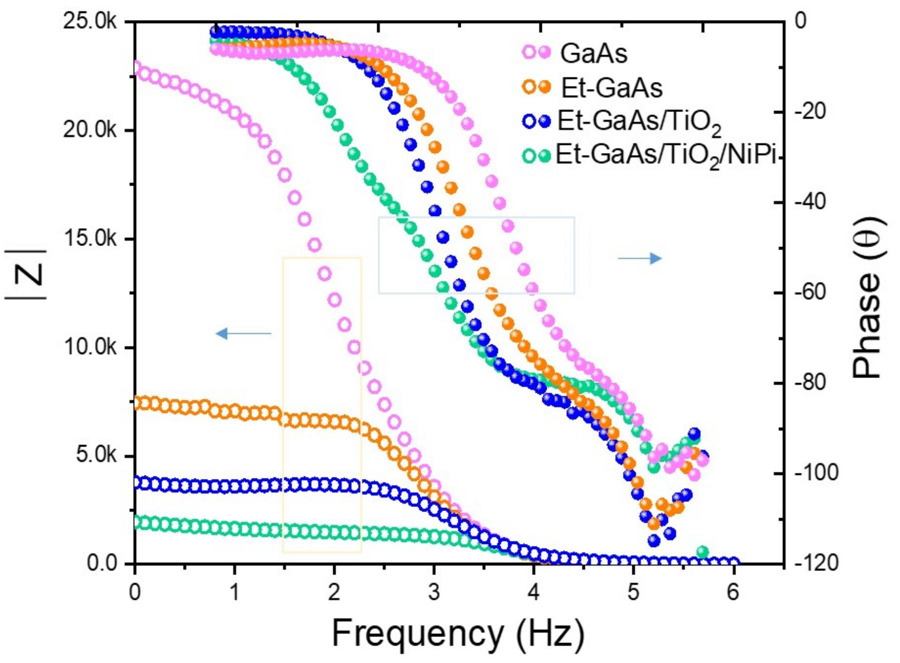

Supplementary Figure 12. Bode plots and magnitude of the impedance verses logarithmic frequency of GaAs, Et-GaAs, Et-GaAs/TiO_2, and Et-GaAs/TiO_2/Ni-Pi films.

Bode plots represent the EIS data consisting of two separate plots including the magnitude of the impedance (|Z|) vs. frequency (logarithmic scale) and the phase angle (θ) vs. frequency (logarithmic scale), seen in Supplementary Fig. 12. The Bode plots can show the frequency range where certain photoelectrochemical processes dominate, such as charge transfer or mass transport. Magnitude of the impedance (|Z|) vs. frequency plot helps to identify the overall impedance and its variation with frequency. In general, a high impedance at low frequencies often indicates the diffusion or mass transport limitations, while a lower impedance at higher frequencies suggests the faster charge transfer process. The phase angle plot distinguish between capacitive and resistive behaviors and determine the dominant processes at different frequency ranges. That is, a phase angle near 0° indicates the resistive behavior (*e.g.,* charge transfer), while a phase angle near -90° suggests capacitive behavior (e.g., double-layer charging or mass transport). As the frequency increases, the impedance magnitude decreases, and the phase angle starts to shift. When the phase angle reaches -45°, it typically indicates the onset of charge transfer processes, such as electron transfer between the photoelectrode and the electrolyte.

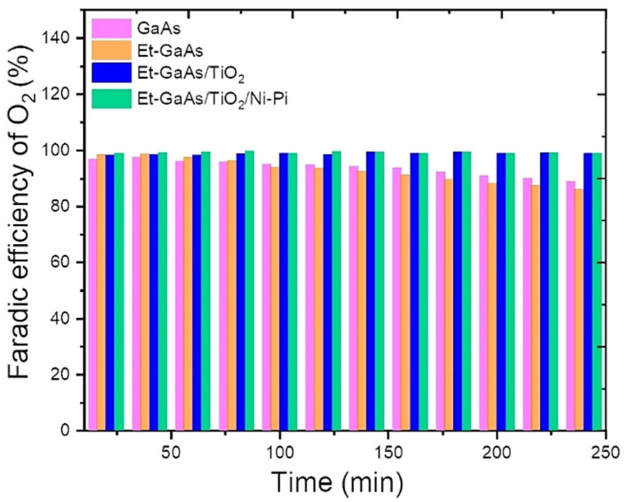

Supplementary Figure 13. O_2 evolution rate corresponding the FE (%) of GaAs, Et-GaAs, Et-GaAs/TiO_2 and Et-GaAs/TiO_2/NiPi photoanode as a function of time.

As shown in Supplementary Fig. 13, the FE(%) of GaAs film decreased over time, subsequently corresponding to the photo-corrosion effect. In the case of photo-corrosion, the excited electrons or holes can react with the electrolyte or the semiconductor itself, leading to the formation of reactive byproducts such as hydroxyl radicals or other oxygen species or defects that can reduce the PEC performance. These side reactions (e.g., non-conductive native oxide or non-faradic charging process at the interface etc.) induce to a side current that competes with the desired photocurrent, leading to lower FE. Also, this dark current can be caused by the formation of a potential barrier at the interface between the corroded and non-corroded parts of the material. The potential barrier acts as a barrier for the electron flow, making the photocurrent pathway around the corroded area.

In the case of Et-GaAs film, the FE is significant at the initial reaction time due to the high surface area effect. As time is going, the etched GaAs photoanodes are vulnerable to photo-corrosion, which can degrade their PEC performance over time. Etching can increase the surface area of the GaAs photoanode, which provides more sites for the electrolyte to react with the photoelectrode, leading to an increased susceptibility to photo-corrosion. Therefore, minimizing the photo-corrosion effect is important for improving the PEC performance and stability. However, there is no meaning degradation observed in the photoelectrodes such as Et-GaAs/TiO_2, and Et-GaAs/TiO_2/NiPi photoanodes due to the surface passivation effect, surely demonstrating the stable PEC performance and co-catalyst boosts the rapid charge transfer reaction at the surface interface.

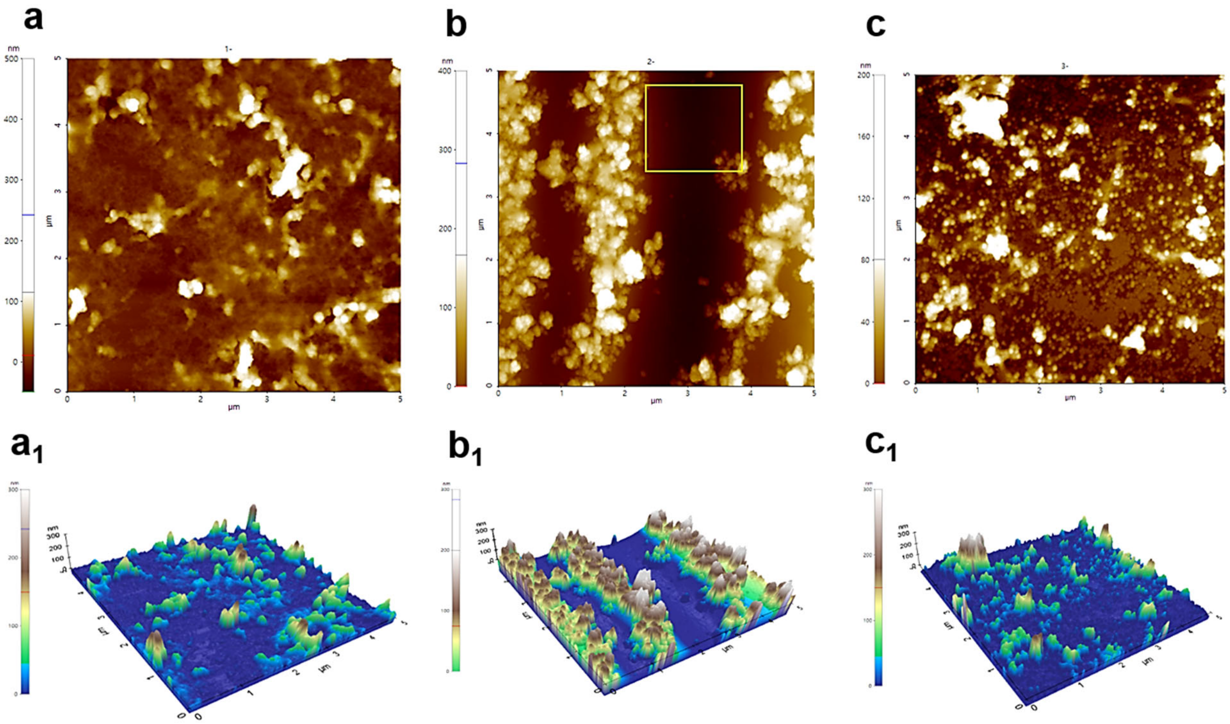

Supplementary Figure 14. Surface 2D and 3D topography images of photoanodes. (a, a_1) Et-GaAs/TiO_2 (before stability), (b, b_1) Et-GaAs/TiO_2 (after stability), (c, c_1) Et-GaAs/TiO_2 Ni-Pi (after stability) films.

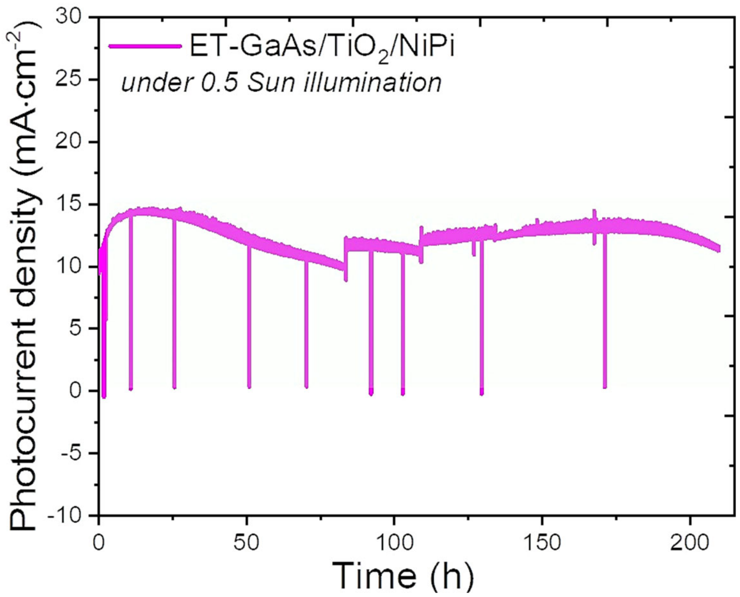

Supplementary Figure 15. Long-term stability test of Et-GaAs/TiO_2/Ni-Pi films collected at 1.23 V_{RHE} under 0.5-sun illumination.

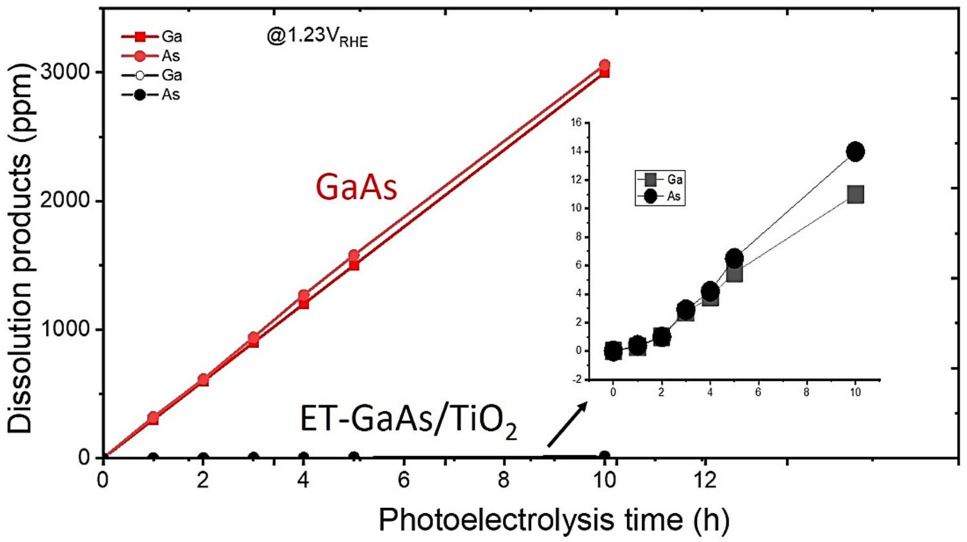

Supplementary Figure 16. ICP-OES analysis of dissolved elements in the electrolyte after stability testing of GaAs and ET-GaAs/TiO_2 films, with the inset representing the magnified view of ET-GaAs/TiO_2.

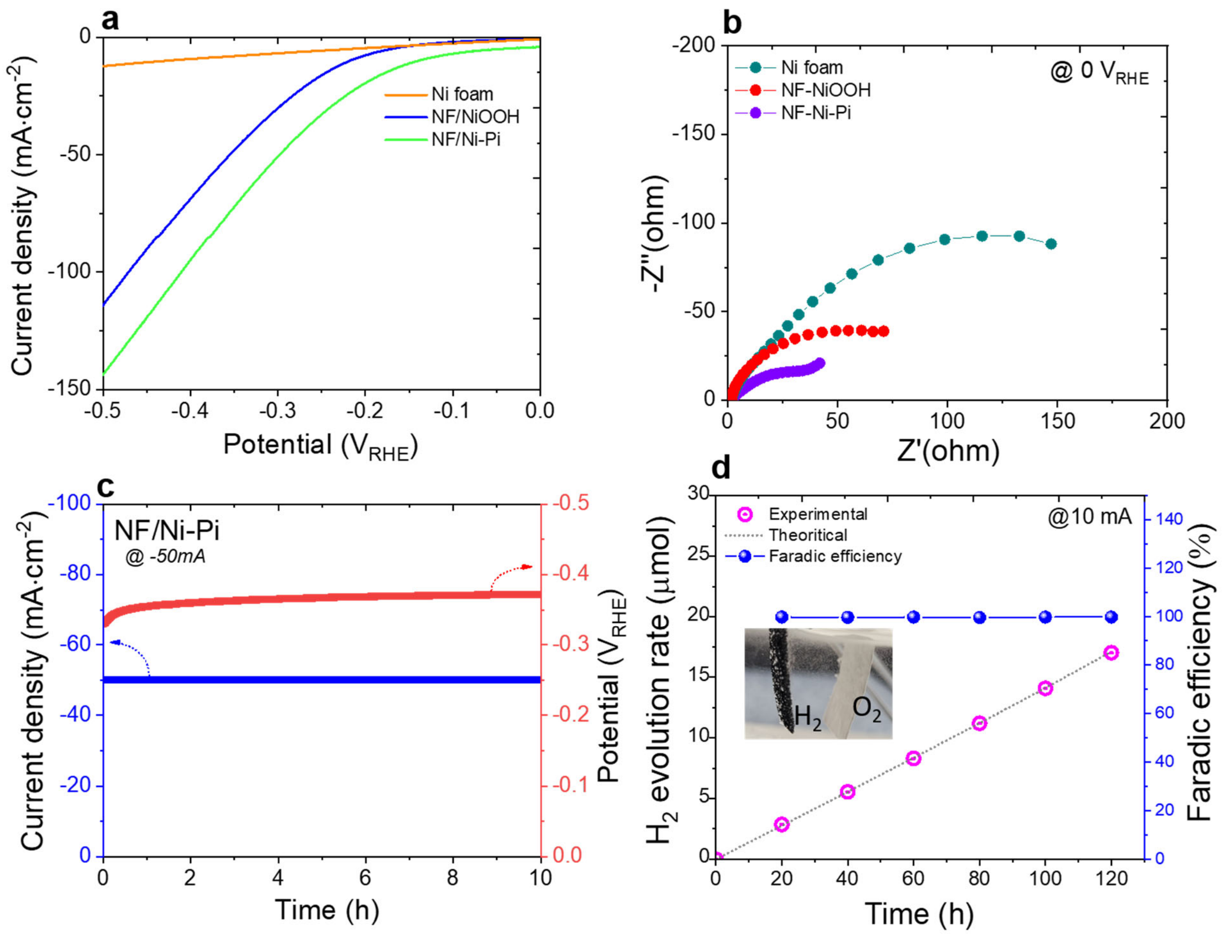

Supplementary Figure 17. Electrocatalytic hydrogen evolution measured using the nickel foam substrate, NF/NiOOH, and NF/Ni-Pi at 1M KOH. (a) LSV polarization curves, (b) Electrochemical impedance Nyquist plot measured at water reduction potential, (c) Chronopotentiometric measurement of the electrocatalyst at -50 mA·cm^{-2}, (d) Quantitative H_2 evolution rate measured by GC at constant applied current.

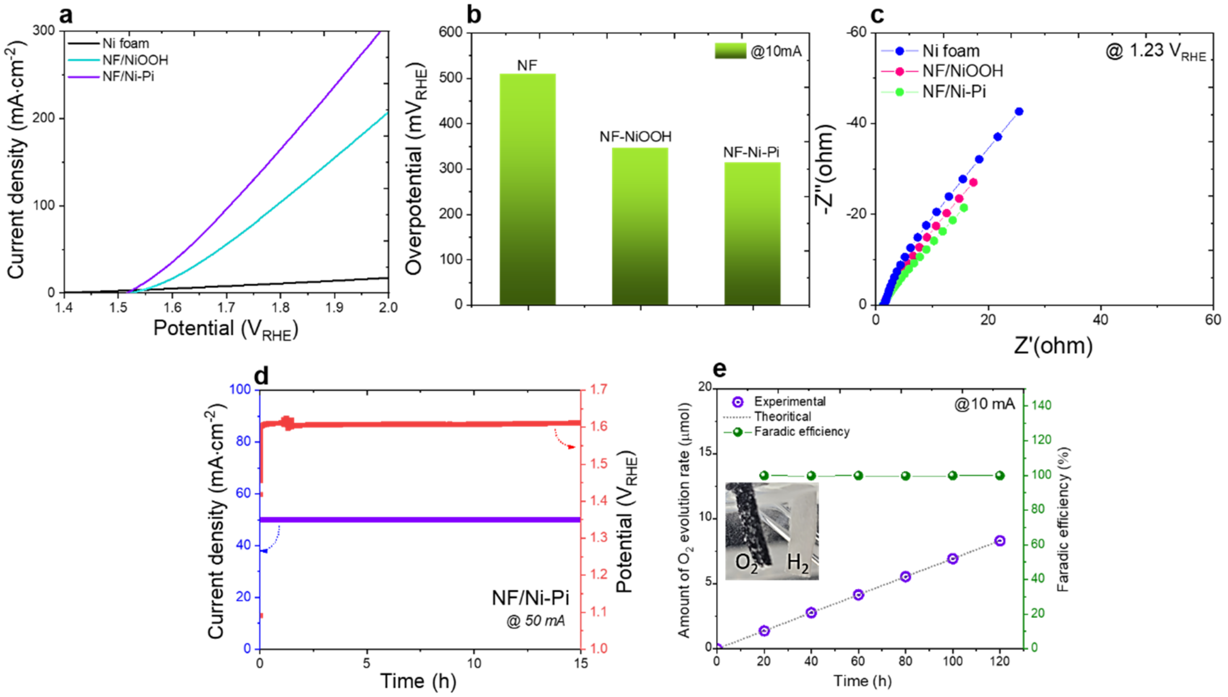

Supplementary Figure 18. Electrocatalytic oxygen evolution measured using nickel foam substrate, NF/NiOOH, and NF/Ni-Pi at 1M KOH. (a) LSV polarization curves. (b) Overpotential of catalyst at 10 mA. (c) Electrochemical impedance Nyquist plot measured at water oxidation potential. (d) Chronopotentiometric measurement of the electrocatalyst at 50 mA·cm^{-2}, revealing the good stability. (e) Quantitative O_2 evolution rate measured by GC at constant applied current.

Supplementary Fig. 17 shows the HER activities of nickel phosphate (Ni-Pi) in alkaline solution, delivering a current density of 10 mA·cm^{-2} at an extremely small overpotential (η_{10}) of 120 mV and extraordinary stability. The FE(%) to induce to the desired products is evolved without any other side reactions. Similarly, Supplementary Fig. 18 demonstrates the efficient OER activity of Ni-Pi, facilitating a current density of 10 mA·cm^{-2} at an extremely small η_{10} of 300 mV and shows excellent electrocatalytic stability in prolonged water electrolysis, meaning that it is highly active to promote the bi-functional properties for the solar water splitting.

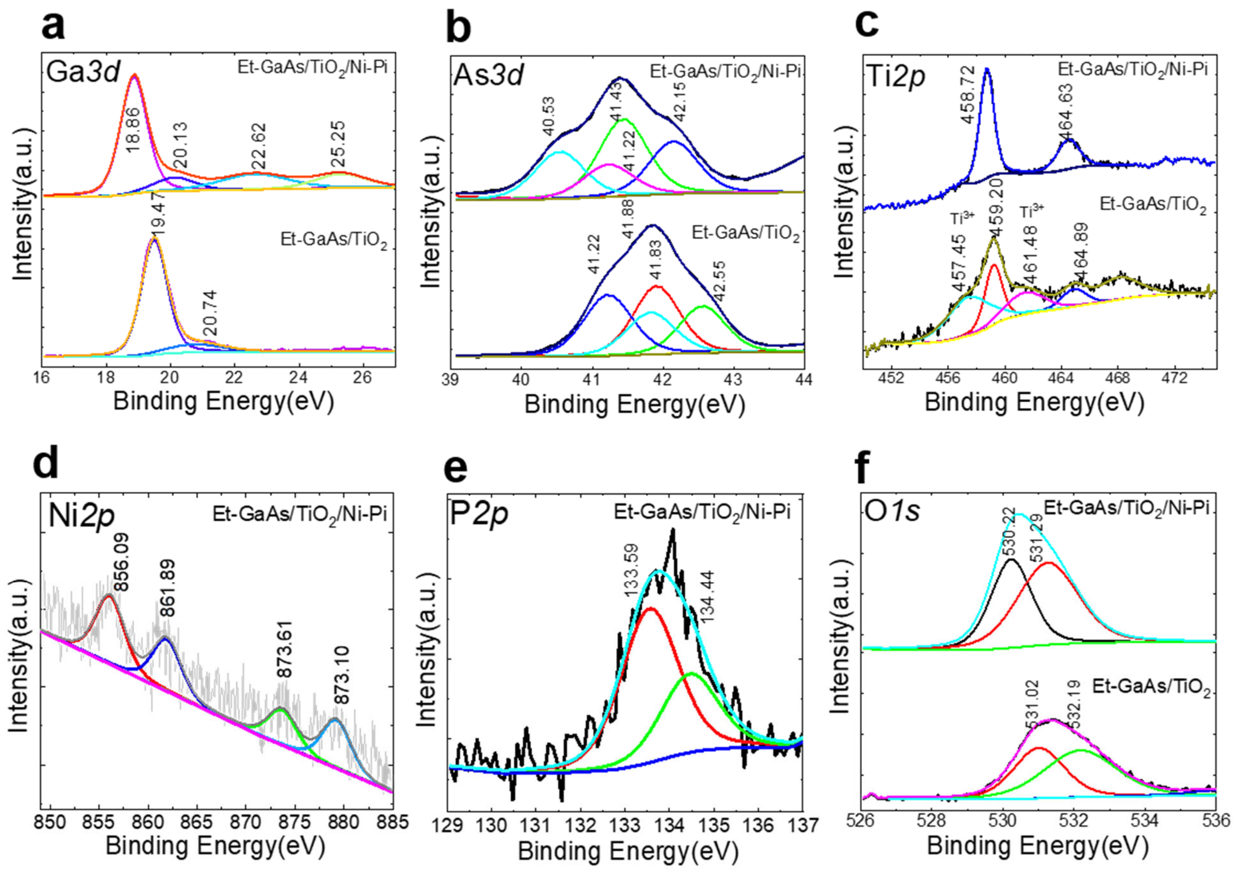

Supplementary Figure 19. (a–f) Core-level XPS spectra of Ga*3d*, As*3d*, Ti*2p*, Ni*2p*, P*2p*, and O*1s* in GaAs, Et-GaAs/TiO_2, and Et-GaAs/TiO_2/Ni-Pi photoanodes after stability testing.

In order to understand the change of chemical states in the photoanode after PEC stability testing, the core-level XPS spectra of Ga*3d*, As*3d*, Ti*2p*, Ni*2p*, P*2p*, and O*1s* in the GaAs/TiO_2 and GaAs/TiO_2/Ni-Pi films were compared and are shown in Supplementary Fig. 19(a–f). The full survey spectrum confirms the presence of Ga, As, Ti, O, Ni, and P elements. The core-level XPS spectrum of Ga*3d* in Supplementary Fig. 19(a) was resolved into the binding energies of 19.49 eV and 18.86 eV from Ga-As and 20.74 eV and 20.13 eV from the oxidized Ga species in the GaAs/TiO_2 and GaAs/TiO_2/Ni-Pi films. A further peak is found in the binding energy of 22.62 eV in GaAs/TiO_2/Ni-Pi film, ascribed to Ga-O-P bonding, and this further evidenced in FIB-TEM with EDX line spectra (Figure 5(e)). Supplementary Fig. 19(b) shows the core-level As*3d* spectra, showing two symmetric peaks. These peaks can be deconvoluted into the binding energies of 41.22 eV and 40.53 eV from As-Ga bonding, 41.83 eV and 41.22 eV from As^0, respectively. And, the binding energies of 41.88 eV and 41.43 eV are coming from As^{+3} state at the form of As_2O_3 and 42.55 eV and 42.15 eV, coming from As^{+5} state at the form of As_2O_5, in the GaAs/TiO_2 and

GaAs/TiO_2/Ni-Pi film, respectively. The core-level XPS spectra of Ti*2p* (Supplementary Fig. 19(c)) can be deconvoluted into two different peaks centered at binding energies of 457.45 eV (Ti$2p_{3/2}$) and 461.48 eV (Ti$2p_{1/2}$), which are assigned to the Ti^{+3} oxidation state in TiO_2. In the GaAs/TiO_2 photoanode, an intense and broad peak can be observed after stability testing. Thus, it clearly shows that the oxidation state is manipulated during the long-term stability test, and the peak corresponding to the +4 oxidation state in the spectrum is shifted toward a higher binding energy compared to the initial sample, due to the higher oxidation potential in the alkaline environment. Surprisingly, there is no evidence of changes to the core-level Ti*2p* spectra in the GaAs/TiO_2/Ni-Pi film, implying that the Ni-Pi deposition is well-developed around the TiO_2 layer to avoid electrolyte contact. This phenomenon is well-matched with the stability test (Figure 5(a)).

The high-resolution spectrum of Ni*2p* in GaAs/TiO_2/Ni-Pi film can be deconvoluted into Ni^{3+} (856.09 eV and 873.10 eV) and satellite peaks (861.89 and 873.10 eV), displayed in Supplementary Fig. 19(d). The continuous oxidation under light illumination under 1 M NaOH strongly interacts with the Ni-Pi layer, and notably, the lower oxidation state of Ni peaks observed in the initial film (Supplementary Fig. 3(d)) disappeared. The formation of nickel oxyhydroxide (NiOOH) species is associated with the redox reaction between Ni^{2+} and Ni^{3+} ions. The redox reaction typically occurs at the surface of the Et-GaAs/TiO_2/Ni-Pi during the PEC stability test, following the equation S3:

$$Ni(OH)_2(s) + OH^-(aq) \leftrightarrow NiOOH(s) + H_2O(l) + e^- \qquad (S3)$$

Additionally, the redox reaction between Ni^{2+} and Ni^{3+} in the formation of NiOOH can also contribute to the self-healing properties and facilitate better electronic conductivity in the Et-GaAs/TiO_2/Ni-Pi film by improving charge transfer kinetics at photoanode/electrolyte interface. Also, the formation of more active sites on the electrocatalyst surface results in faster reaction rates and lower overpotentials for increasing the energy conversion efficiency of the PEC device. As a conclusion, NiPi in the Et-GaAs/TiO_2 photoanode film is strong and significant even after the long-term stability test under harsh condition.

Furthermore, from the core-level P*2p* spectra (Supplementary Fig. 19(e)), it can be seen that the binding energies of 133.59 and 134.44 eV are consistent with those of Ni$2p_{3/2}$ and Ni$2p_{1/2}$ in $Ni_3(PO_4)_2$, assigned to the spin-orbit doublets characteristic of the Ni^{3+} state. Interestingly, there is no change in the peak intensity of the core-level P*2p* spectrum. A lower BE migration is observed, compared to before stability testing (Supplementary Fig. 6(e)). This can be associated with the

formation of Ga-P after the long-term stability test. To delicately survey the P element after long-term stability testing, FIB-TEM analysis integrated with EDX mapping and in-depth line mapping was performed, as shown in Figure 5(f). We found that the PO_4^{3-} ions combined with Ga to form a Ga-O-P layer on the surface after long-term stability testing. Furthermore, in the case of the O1s spectra of GaAs/TiO_2 and GaAs/TiO_2/Ni-Pi film (Supplementary Fig. 19(f)), two major component peaks at the binding energies of (529.37 and 530.80 eV) and (529.37 and 530.80 eV) come from the metal hydroxides (M-OH), water (H_2O_{ad}) groups, and the lattice species ((O_L) and $(PO_4)^{3-}$), respectively.

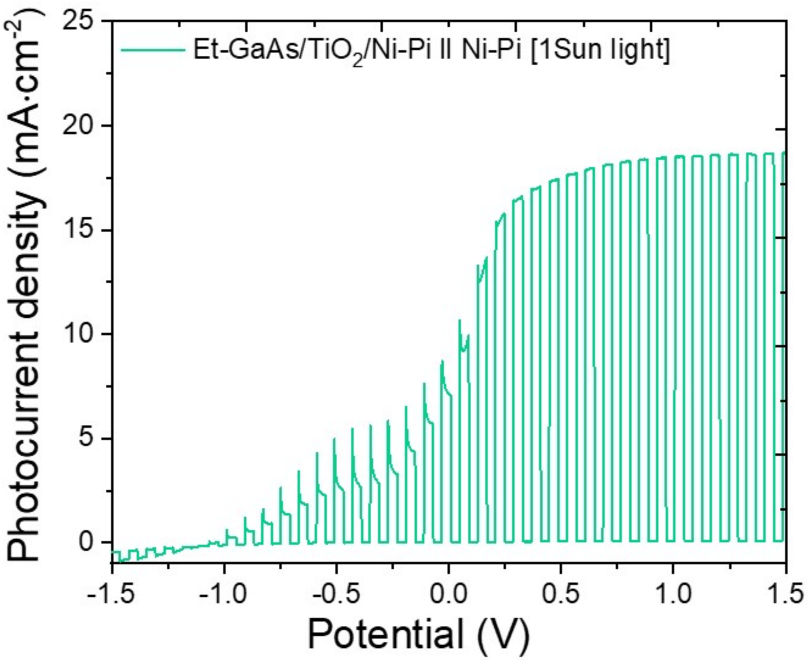

Supplementary Figure 20. LSV curve of Et-GaAs/TiO_2/Ni-Pi‖Ni-Pi electrocatalyst in the tandem cell configuration under chopped illumination.

In the two-electrode configuration, the solar-to-hydrogen (STH) conversion efficiency was calculated using the equation S4.[12] Based on the linear sweep voltammetry measurement of our best-performed Et-GaAs/TiO_2/Ni-Pi‖Ni-Pi nanowire photocathode, demonstrated in Supplementary Fig. 20, an STH efficiency is determined to be ~9.5%. In addition, with using the equation below, it is assumed that the faradaic efficiency is unity, which has been demonstrated in Supplementary Fig. 20.

$$\text{STH}=\left(\frac{Jph(mA/\ cm^2)\times(1.23V-Vapp)}{Pin\ (\frac{mw}{cm^2})}\right)AM1.5G \qquad \text{(S4)}$$

where J_{ph} is the measured photocurrent in a two-electrode configuration, V_{app} represents the external electric bias (V vs. NiPi counter electrode), and P_{in} is power density of the incident light is 100 mW·cm^{-2}.

The recently updated PEC performance on GaAs based PEC system and tandem cell is summarized at Table S2, based on the fabrication method, photocurrent density (J_{ph}), electrolyte, and stability.

Supplementary Table 2. Summary on the GaAs based PEC performance in terms of fabrication method, photocurrent density (J_{ph}), electrolyte, and stability.

GaAs based PEC cell					
Photoanode	Fabrication	J_{ph} ($mA \cdot cm^{-2}$)	Electrolyte	Stability	Ref.
GaAs/TiO_2/Ni core-shell nanorods	GLAD	12.87	1M NaOH	3h	13
GaAs/Ga_2S_3heterostructure	Sulfurization	12.87	0.1M Na_2SO_4	500s	14
GaAs(100)/single layer of graphene	Vertical gradient-freeze method, CVD	25.8	CH_3CN-$Fc^{+/0}$	8h	15
GaAs/Polythiophene	Electrodeposition	3.52	0.1M $K_3Fe(CN)$, 0.25M $K_4Fe(CN)_6$	100h	16
GaAs NW arrays/NiO_x	MOCVD, E-beam Lithography, ALD	11.1	Fc/Fc^+ redox couple	-	17
GaAs/TiO_2/Ni	ALD	14.3	1M KOH	100h	18
GaAs/Ni–B	MOCVD, Photo-assisted Electrodeposition	20	0.1M KOH	22h	19
GaAs/Ni	Electrodeposition	9.2	$K_3Fe(CN)_6$/$K_4Fe(CN)_6$	300s	20
GaAs/a-TiO_2/NiO_x	MOCVD, ALD, Sputtering	8.3	1M KOH	600h	21
TiN/N-TiO_2/ITO/GaAs	RF sputtering, NH_3 Plasma, PECVD	17.82	1M KOH	6h	22
GaAs/Ir	Spin-coating	18	1M H_2SO_4	-	23
Et-GaAs/TiO_2/Ni-Pi	Electrochemical etching, Spin-coating, and Electrodeposition	25	1M NaOH	~110h (1-Sun) ~ 200h (0.5 Sun)	This work
GaAs-based Tandem cell					
Tandem cell	Fabrication	Configuration	STH (%)	stability	Ref.
GaAs/InGaAsP‖WO_3/$BiVO_4$	Electrochemical deposition	PV–PEC	8.1%.	1h	24
InGaP‖GaAs	MOCVD, Electrodeposition	PEC	9%	150 h	25
$In_{0.25}Ga_{0.75}N$‖Pt	plasma-assisted molecular beam epitaxy	PEC–EC	3.4%	~300 h	26
$BiVO_4$‖p^+n-$GaAs_{1-x}Pt_x$	Spray pyrolysis	PV–PEC	1.8%	3h	27
Et-GaAs/TiO_2/Ni-Pi‖Ni-Pi@Ni foam	Electrochemical etching, Spin-coating, and Electrodeposition	PEC-EC	9.5%	~35h	This work

Supplementary References:

1. C. Jiang, J. Wu, S.J. Moniz, D. Guo, M. Tang, Q. Jiang, S. Chen, H. Liu, A. Wang, T. Zhang, J. Tang, Stabilization of GaAs photoanodes by in situ deposition of nickel-borate surface catalysts as hole trapping sites. Sustain. Energy Fuels. **3(3)** (2019) 814-822.
2. S. Pishgar, M.C. Mulvehill, S. Gulati, G.U. Sumanasekera, J. M. Spurgeon, Investigation of n-GaAs Photoanode Corrosion in Acidic Media with Various Thin Ir Cocatalyst Layers. ACS Appl. Energy Mater. **4(10)** (2021) 10799-10809.
3. A.J. Henegar, A.J. Cook, P. Dang, T. Gougousi, Native oxide transport and removal during atomic layer deposition of TiO_2 films on GaAs (100) surfaces. ACS Appl. Energy Mater. **8(3),** (2016) 1667-1675.
4. J. Zheng, Y. Lyu, R. Wang, C. Xie, H. Zhou, S.P. Jiang, S. Wang, Crystalline TiO_2 protective layer with graded oxygen defects for efficient and stable silicon-based photocathode. Nat. Commun. **9(1)** (2018) 1-10.
5. J. Qiu, G. Zeng, M. Ge, S. Arab, M. Mecklenburg, B. Hou, C. Shen, A.V. Benderskii, S. B. Cronin, Correlation of Ti^{3+} states with photocatalytic enhancement in TiO_2-passivated p-GaAs. Journal of Catal. **337** (2016) 133-137.
6. Y. Pan, Y. Liu, J. Zhao, K. Yang, J. Liang, D. Liu, W. Hu, D. Liu, Y. Liu, C. Liu, Monodispersed nickel phosphide nanocrystals with different phases: synthesis, characterization and electrocatalytic properties for hydrogen evolution. J. Mater. Chem. A, **3(4)** (2015) 1656-1665.
7. Y. Guo, J. Tang, Z. Wang, Y. Sugahara, Y. Yamauchi, Hollow porous heterometallic phosphide nanocubes for enhanced electrochemical water splitting. Small, **14(44)** (2018) 1802442.
8. R. Li, B. Hu, T. Yu, H. Chen, Y. Wang, Song, S. Insights into correlation among surface-structure-Activity of cobalt-derived pre-catalyst for oxygen evolution reaction. Adv. Sci. **7(5)** (2020) 1902830.
9. J.A. Cecilia, A. Infantes-Molina, E. Rodríguez-Castellón, A. Jiménez-López, A novel method for preparing an active nickel phosphide catalyst for HDS of dibenzothiophene. Journal of Catal. **263(1)** (2009) 4-15.
10. R. Ye, P. del Angel-Vicente, Y. Liu, M.J. Arellano-Jimenez, Z. Peng, T. Wang, Y. Li, B.I. Yakobson, S.H. Wei, M.J. Yacaman, J.M. Tour, High-performance hydrogen evolution from $MoS_{2(1-x)}P_x$ solid solution. Adv. Mater., **28(7)** (2016) 1427-1432.
11. Y. Xu, R. Ahmed, J. Zheng, E.R. Hoglund, Q. Lin, E. Berretti, A. Lavacchi, G. Zangari, Photoelectrochemistry of Self-Limiting Electrodeposition of Ni Film onto GaAs. Small. **16(39)** (2020) 2003112.
12. Y. Wang, Y. Wu, J. Schwartz, S. H. Sung, R. Hovden, Z. Mi, A single-junction cathodic approach for stable unassisted solar water splitting. Joule. **3(10)** (2019) 2444-2456.
13. Alqahtani, M., Kafizas, A., Sathasivam, S., Ebaid, M., Cui, F., Alyamani, A., & Wu, J. A Hierarchical 3D TiO_2/Ni Nanostructure as an Efficient Hole-Extraction and Protection Layer for GaAs Photoanodes. ChemSusChem, **13(22)** (2020) 6028-6036.

14. Liu, H. F., Antwi, K. A., Chua, C. S., Huang, J., Chua, S. J., & Chi, D. Z. (2014). Epitaxial synthesis, band offset, and photoelectrochemical properties of cubic Ga_2S_3 thin films on GaAs (111) substrates. ECS Sol. State Lett., **3(11)** (2014) P131.
15. Yang, F., Nielander, A. C., Grimm, R. L., & Lewis, N. S. Photoelectrochemical behavior of n-type GaAs (100) electrodes coated by a single layer of graphene. J. Phys. Chem. C **120(13)** (2016) 6989-6995.
16. Horowitz, G., & Garnier, F. Long-Term Stabilization of Polythiophene-Protected n-GaAs Photoanodes in Aqueous Solution. J. Electrochem. Soc. **132(3)** (1985) 634.
17. Zeng, J., Xu, X., Parameshwaran, V., Baker, J., Bent, S., Wong, H. S. P., & Clemens, B. (2018). Photoelectrochemical water oxidation by GaAs nanowire arrays protected with atomic layer deposited NiOx electrocatalysts. J. Electron. Mater., 47, (2018) 932-937.
18. Hu, S., Shaner, M. R., Beardslee, J. A., Lichterman, M., Brunschwig, B. S., & Lewis, N. S. Amorphous TiO_2 coatings stabilize Si, GaAs, and GaP photoanodes for efficient water oxidation. Science, **344(6187)** (2014) 1005-1009.
19. Jiang, C., Wu, J., Moniz, S. J., Guo, D., Tang, M., Jiang, Q., Tang, J. Stabilization of GaAs photoanodes by in situ deposition of nickel-borate surface catalysts as hole trapping sites. Sustain. Energy Fuels, **3(3)** (2019) 814-822.
20. Xu, Y., Ahmed, R., Zheng, J., Hoglund, E. R., Lin, Q., Berretti, E., Zangari, G. Photoelectrochemistry of Self-Limiting Electrodeposition of Ni Film onto GaAs. Small, **16(39)** (2020) 2003112.
21. Shen, X., Yao, M., Sun, K., Zhao, T., He, Y., Chi, C. Y., Hu, S. Defect-tolerant TiO_2-coated and discretized photoanodes for > 600 h of stable photoelectrochemical water oxidation. ACS Energy Lett., **6(1)** (2020) 193-200.
22. Choi, K., Bang, J., kyu Moon, I., Kim, K., Oh, J. (2020). Enhanced photoelectrochemical efficiency and stability using nitrogen-doped TiO_2 on a GaAs photoanode. J. Alloys Compd., **843** (2020) 155973.
23. Pishgar, S., Mulvehill, M. C., Gulati, S., Sumanasekera, G. U., Spurgeon, J. M. Investigation of n-GaAs Photoanode Corrosion in Acidic Media with Various Thin Ir Cocatalyst Layers. ACS Appl. Energy Mater., **4(10)** (2021) 10799-10809.
24. Kosar, S., Pihosh, Y., Turkevych, I., Mawatari, K., Uemura, J., Kazoe, Y., Kitamori, T. Tandem photovoltaic–photoelectrochemical GaAs/InGaAsP–WO_3/$BiVO_4$ device for solar hydrogen generation. Jpn. J. Appl. Phys., **55(4S)** (2016) 04ES01.
25. Varadhan, P., Fu, H. C., Kao, Y. C., Horng, R. H., & He, J. H. An efficient and stable photoelectrochemical system with 9% solar-to-hydrogen conversion efficiency via InGaP/GaAs double junction. Nature Comm., **10(1)** (2019) 5282.
26. Wang, Y., Wu, Y., Schwartz, J., Sung, S. H., Hovden, R., & Mi, Z. A single-junction cathodic approach for stable unassisted solar water splitting. Joule, **3(10)** (2019) 2444-2456.
27. Lawrence, D. J., Smith, B. L., Collard, C. D., Elliott, K. A., Fakhoury, K. L., Mangold, J. D., Soyka, A. N. Monolithically-integrated $BiVO_4$/p^+-n $GaAs_{1-x}P_x$ tandem photoanodes capable of unassisted solar water splitting. Int. J. Hydrog. Energ., **46(2)** (2021) 1642-1655.
